# Plant-Based vs. Pork Sausages: Protein Nutritional Quality and Antioxidant Potential in the Bioaccessible Fraction

**DOI:** 10.3390/foods14244271

**Published:** 2025-12-11

**Authors:** Narigul Khamzaeva, Bettina Hieronimus, Christina Kunz, Larissa E. Pferdmenges, Karlis Briviba

**Affiliations:** 1 Department of Physiology and Biochemistry of Nutrition, Max Rubner-Institut—Federal Research Institute of Nutrition and Food, 76131 Karlsruhe, Germany; narigul.khamzaeva@mri.bund.de (N.K.); christina.kunz@mri.bund.de (C.K.); karlis.briviba@mri.bund.de (K.B.); 2 Department of Nutritional Behaviour, Max Rubner-Institut—Federal Research Institute of Nutrition and Food, 76131 Karlsruhe, Germany; larissa.pferdmenges@mri.bund.de

**Keywords:** DIAAS, in vitro protein digestibility, plant-based sausages, antioxidant potential

## Abstract

The sales volume and consumption of plant-based meat substitutes is steadily increasing. Since meat and meat products are an important protein source, this study focused on the nutritional protein quality (Digestible Indispensable Amino Acid Score (DIAAS)) of alternative sausages based on soy, wheat, and a wheat-soy blend in comparison to a pork-based sausage using the tiny-TIMsg gastrointestinal model. The protein digestibility of all products ranged from 80.8% (soy sausage) and 87.1 to 89.0% (other sausages). The highest DIAAS values were obtained for pork sausage limited by leucine (116%). Soy sausage was limited in sulfur-containing amino acids (86%). Wheat and wheat-soy sausages were limited by lysine (33% and 41%, respectively). The antioxidant activity of the bioaccessible fractions revealed a higher antioxidative potential of the plant-based sausages. While plant-based sausages offer comparable protein digestibility and superior antioxidant capacity, their significantly lower DIAAS values underscore the potential for formulation strategies that consider nutritional aspects.

## 1. Introduction

The production and consumption of meat and other animal-based products place a significant burden on the environment [1]. These environmental concerns, along with increasing awareness of animal welfare, are leading many consumers to reduce their intake of animal-based foods [2]. In 2025, more than 50% of Western European consumers reported limiting their consumption of red meat [3]. The decrease in meat consumption is paralleled by a growing popularity of alternatives for meat and meat products [4]. German companies significantly increased their production of meat alternatives in 2023, showing a 16.6% rise over 2022 and a more than twofold increase (+113.8%) when compared to 2019 [5].

Traditionally, meat is considered a valuable source of high-quality protein and essential nutrients. Protein quality is assessed using several criteria: the total protein content, the amino acid composition, and the bioaccessibility of the protein, with a specific focus on the digestibility and availability of indispensable amino acids (IAAs) [6]. These factors collectively determine the nutritional value of a dietary protein source. The protein quality of plant-based products can differ markedly from that of the meat-based products they are intended to replace [7]. The content of IAAs varies depending on the plant protein source. For instance, legumes (like soy) are typically limited by sulfur-containing amino acids (methionine and cysteine), while cereals (like wheat) are usually limited by lysine. The bioavailability of IAAs is further influenced by the structural characteristics of plant proteins as their lower solubility and greater molecular complexity can negatively affect their digestibility [7,8]. Additionally, many plant-based ingredients contain so-called antinutritional factors such as phytates, tannins, or protease inhibitors [9,10]. These compounds can impair protein digestion by inhibiting digestive enzymes or by binding to amino acids and minerals, thereby reducing their absorption in the gut [9].

However, secondary plant compounds have been associated with beneficial effects on health outcomes [9]. These beneficial effects stem, amongst other mechanisms, from the antioxidant capacity of compounds like phenolic acids, flavonoids, and other antioxidants which are also present in plant-based meat analogs [11,12,13]. Studies on antioxidant capacity are usually based on unprocessed plant products or extracts [14], but less is known about the antioxidant effects of processed foods, like plant-based sausage analogs.

Although the total protein and IAA content, as well as the antioxidant capacity, can be chemically analyzed, such measurements alone are insufficient to assess the true nutritional value of a food item. To that end, digestibility and bioaccessibility must be considered, as these are physiological processes that require in vivo analyses for proper evaluation [15,16]. Traditionally, animal studies involving the collection of ileal chyme have been used to determine amino acid digestibility. In alignment with the 3R principle (Reduce, Refine, Replace), alternative in vitro methods have been developed to minimize the use of animals in nutritional research. One of the most advanced and extensively validated system is TNO’s dynamic gastrointestinal model (tinyTIMsg) [17]. This model simulates the conditions of the human gastrointestinal tract, including dynamic pH changes, peristaltic mixing, gastric emptying, and the presence of digestive enzymes and bile. By mimicking the conditions of the stomach and small intestine, the tinyTIMsg enables detailed assessment of protein digestibility and the bioaccessibility of amino acids under standardized, reproducible conditions [17,18]. The Digestible Indispensable Amino Acid Score (DIAAS), recommended by the FAO in 2013 [6], assesses protein quality by considering both the total protein content and, crucially, the digestibility and bioaccessible amino acid composition of a food item, specifically the digestibility of each indispensable amino acid at the end of the small intestine compared to a reference protein. While traditional indices such as the Chemical Score or Protein Efficiency Ratio have been widely employed in the past, they typically fail to account for the specific ileal digestibility of individual amino acids. Consequently, this study focuses exclusively on the DIAAS as the most advanced and physiologically relevant metric for human nutrition, rendering these older proxy measures redundant for the scope of this investigation.

As a widely consumed processed meat product in Germany, scalded sausages (finely ground, emulsified, and heat-treated at 70–80 °C) serve as a relevant reference for evaluating plant-based meat alternatives. In this study, a traditional pork-based scalded sausage was compared to three plant-based alternatives formulated with soy, wheat, and a wheat-soy blend in order to assess their nutritional protein quality. Protein digestibility and DIAAS were determined after in vitro digestion using the dynamic tiny-TIMsg model.

Additionally, the antioxidant capacity of the bioaccessible fraction was analyzed with three complementary assays: the Ferric Reducing Ability of Plasma (FRAP) assay, the Oxygen Radical Absorbance Capacity (ORAC) assay, and the Trolox Equivalent Antioxidant Capacity (TEAC) assay. Previous work utilizing this methodology has successfully characterized the protein quality of liquid food systems, such as various plant-based milk alternatives [19]. However, the impact of highly processed, solid, emulsified matrices, as present in the scalded sausages studies here, on protein quality and bioaccessibility remains largely unknown.

By integrating these analyses, the aim of this work was to provide a holistic understanding of the nutritional and functional properties of plant-based sausages compared to their pork-based counterpart. Understanding their digestible protein quality and bioaccessible antioxidant capacity is relevant for public health and product reformulation, as these foods are gaining substantial market share in many regions.

## 2. Materials and Methods

### 2.1. Test Products

The sampling procedure of this investigation is part of a larger project evaluating the nutritional quality of plant-based meat alternatives (data will be published elsewhere). In brief, the meat alternatives were drawn based on a sampling protocol for the German food market. For this study, only plant-based scalded sausages based on soy, wheat, and a mix of soy and wheat in addition to a pork-based sausage were sampled. These products are ready to eat but are usually heated before consumption. For each category, the most important manufacturers were identified and one product of each category was selected for this study: “Tofiner” tofu sausages Viennese style from Taifun-Tofu (Freiburg, Germany), “Weenies” vegan organic seitan sausages, smoked from TOPAS (Mössingen, Germany), Tofu & Seitan Sausages from Rossmann (Burgwedel, Germany), and “Deutschländer” pork sausages from Meica (Edewecht, Germany). Product details from the packages are presented in Appendix A (Table A1 and Table A2).

### 2.2. Tiny-TIMsg Model and Protein Digestion Settings

The tiny-TIMsg system (The TIM Company, Delft, The Netherlands) was applied for the dynamic in vitro digestion experiments. This system mimics the processes of gastrointestinal digestion and a detailed validation of this model can be found in Bellmann et al. 2016 [20]. Sausages were preheated according to the manufacturer’s instructions and minced. A tiny-TIMsg protocol for protein digestion was followed, based on a meal intake of 150 g of food containing 5–8 g of protein. To achieve a protein content of 6 g per meal, the required product quantities were calculated according to the packaging information (Table A2): 40 g soy sausage, 19 g wheat sausage, 23 g wheat-soy sausage, and 43 g pork sausage. The product was then mixed with the prescribed amounts of electrolyte solutions and finally filled up with water to a total volume of 150 g. The sample was incubated with α-amylase (approximately 1800 U) at 37 °C for 30 s and then introduced into the gastric compartment of the tiny-TIMsg system. The dynamic in vitro digestion was carried out for 5 h and was computer-controlled as described previously [19]. All required reagents, including bile, sodium acetate trihydrate, sodium citrate tribasic dihydrate, α-amylase (from *Bacillus* sp.), lipase (from *Rhizopus oryzae*), porcine pancreatin 4xUSP, porcine pepsin, and bovine trypsin, were procured from Sigma Aldrich (Schnelldorf, Germany). The specific enzyme activities for pepsin, trypsin, and α-amylase were subsequently determined following the established INFOGEST protocol [21]. Blank digestion experiments were performed using double distilled water (150 g) instead of sausages in order to determine the amounts of nitrogen and amino acids from digestion enzymes, bile, and electrolytes. In vitro digestion experiments were performed in triplicate.

### 2.3. Kjeldahl Analysis

Total organic nitrogen in plant- and pork-based sausages, digestion dialysates, and residues were measured using a BÜCHI system (BÜCHI Labortechnik AG, Flawil, Switzerland) as previously described [19]. The following nitrogen-to-protein conversion factors were used to calculate the protein content from total organic nitrogen: for soy—5.38, for wheat—5.30, for wheat-soy—5.32, and for pork—5.17 [22]. The protein content results are based on the wet weight of the products.

### 2.4. Amino Acid Analysis by HPLC

The concentration of 20 proteinogenic amino acids was measured. The methods of sample preparation were based on Rutherfurd and Gilani 2009 [23]. HPLC analysis and the calculation of ileal protein digestibility as well as DIAAS were described in detail by Khamzaeva et al. 2024 [19].

### 2.5. Antioxidant Capacity

Three antioxidant assays were performed using the bioaccessible fractions of all test products, namely FRAP, ORAC, and TEAC assays. FRAP was performed with modifications of the original publication by Benzie & Strain 1996 [24]. Since the tests have to be carried out in an acidic environment, the bioaccessible fractions of sausages were diluted in acetate buffer (pH = 3.6) and precipitations were removed by centrifugation (10,000 rpm, 5 min). The FRAP reagent was prepared with 12.5 mL of pre-warmed acetate buffer (37 °C), 1.25 mL of 20 mM iron (III) chloride, and 1.25 mL of 10 mM TPTZ. In this assay, different concentrations of iron (II) sulfate were used for calibration (0, 60, 120, 240, 300, 450, 600, and 900 µM). The bioaccessible fractions were measured in a linear concentration range (300–1500 µg food protein/mL) in 96-well plates. A total of 60 µL of standard/sample, respectively, was mixed with 240 µL of FRAP reagent and each preparation was measured in duplicate. The plate was placed in a spectrophotometer and the reduction of ferric ions to ferrous ions was determined using a characteristically blue-colored ferrous tripyridyl triazine complex with a maximum absorption at 590 nm after 4 min. The procedure of the ORAC and TEAC assays were described in detail by Khamzaeva et al. 2024 [19]. The bioaccessible fractions were measured in a linear concentration range (15–150 µg food protein/mL for ORAC and 75–1050 µg food prot./mL for TEAC).

### 2.6. Statistical Analysis

Data is presented as mean ± standard deviation (SD). The statistical analysis was performed using analysis of variance (ANOVA) followed by Tukey’s test using SigmaPlot (version 14.0, Systat Software GmbH; Erkrath, Germany). The normality and homogeneity of variance were assessed prior to conducting an ANOVA. No violations were detected. A *p*-value of <0.05 was defined as the threshold for statistical significance.

## 3. Results

### 3.1. Crude Protein Content of Plant- and Pork-Based Sausages (Kjeldahl Method)

The soy-based sausage contained 12.53 ± 0.43 g protein per 100 g, which was comparable to the pork-based sausage (11.38 ± 0.22 g/100 g) as determined by nitrogen analysis using the Kjeldahl method. In contrast, the wheat-based alternatives had approximately double the amount, with 24.80 ± 0.75 g/100 g in the wheat-soy combination and 21.27 ± 0.35 g/100 g in the purely wheat-based variant. All values are slightly lower than the values given by the manufacturers (Appendix A, Table A2).

### 3.2. Amino Acid Profile and Calculated Protein Content of Plant- and Pork-Based Sausages (HPLC)

The concentrations of the 20 proteinogenic amino acids in the plant-based and pork-based sausages, as determined by HPLC analysis, are presented in Table 1. The amino acid data provide insight into the qualitative composition of the protein in each product and allow for a calculated estimation of total protein content. This was performed by summing the concentrations of all individual amino acids and correcting for the loss of a water molecule per peptide bond formed during protein synthesis.

The total amino acid residue content (Σ amino acid residues) closely matched the crude protein values obtained via the Kjeldahl method (see Section 3.1), validating the consistency between both approaches. Among all samples, the wheat-based sausages had the highest protein content, which was calculated as the sum of the individual amino acid residues (27.731 ± 3.250 g/100 g), followed by the wheat-soy variant (21.730 ± 0.542 g/100 g). These values were significantly higher than those of the soy-based (11.973 ± 0.583 g/100 g) and pork-based sausages (12.249 ± 0.693 g/100 g).

In terms of amino acid distribution, glutamic acid was the most abundant amino acid in all products, with particularly high levels in the wheat-based sausage (Table 1). Among the essential amino acids, leucine consistently showed the highest concentrations across all samples. Expressed proportionally, glutamic acid accounted for approximately 33% of total amino acids in wheat sausages and 18–22% in the other products, whereas leucine contributed between 7 and 10% across all samples. The lowest concentrations were observed for tryptophan in the plant-based sausages and for cysteine in the pork sausage. This detailed amino acid profiling not only confirms the quantitative differences in protein content across formulations but also highlights key compositional aspects that may influence the nutritional quality of the products.

### 3.3. Bioaccessible Protein and Amino Acids of Plant- and Pork-Based Sausages

**Figure 1:**
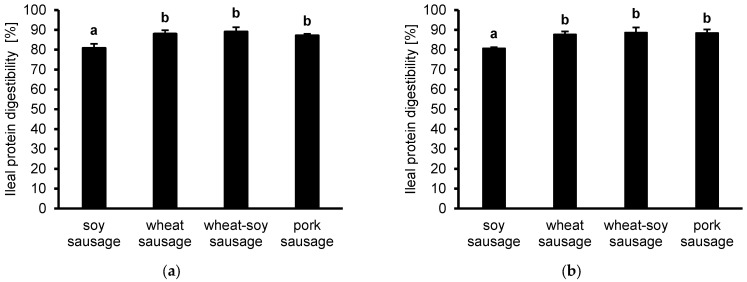
Protein digestibility at the ileal level is presented for sausages (soy, wheat, wheat-soy, and pork) following in vitro simulation using the tiny-TIMsg model. Digestibility was calculated by summing the bioaccessible and non-bioaccessible fractions, quantified via Kjeldahl (**a**) or HPLC (**b**). Data are shown as mean (*n* = 3) ± SD. Tukey’s post hoc test determined statistical variance, where distinct letters mark significant differences (*p* < 0.05).

While the protein digestibility was generally high in all samples, the soy-based sausage exhibited the lowest protein digestibility (80.8 ± 2.2%) compared to all other samples (Figure 1a). In contrast, wheat sausage (88.0 ± 1.9%), wheat-soy sausage (89.0 ± 2.2%), and pork sausage (87.1 ± 0.9%) showed significantly higher digestibility values.

These trends are confirmed in the HPLC-derived amino acid measurements (Figure 1b). Again, soy sausage showed significantly lower digestibility (80.8 ± 0.5%) compared to the wheat- (87.8 ± 1.4), wheat-soy- (88.7 ± 2.6%), and pork-based (88.5 ± 1.7%), which demonstrated comparable and higher digestibility values.

The digestibility of individual amino acids can vary from the overall protein digestibility. Digestibility of IAAs is especially of interest as this provides more precise data on the nutritional quality of the particular food. Therefore, the digestibility of the IAAs is given in Table 2. For the soy-based sausage, methionine showed the lowest and lysine the highest digestibility with 74.8 ± 7.4% and 86.4 ± 1.6%, respectively. These values differ slightly from the overall protein digestibility of 80.8 ± 2.2%, which by definition is calculated based on the sum of the bioaccessible and non-bioaccessible fractions determined via Kjeldahl analysis (nitrogen determination). Individual IAA digestibility was comparable in the other three products, ranging between 78.4 ± 6.0 (met, wheat sausage) and 91.9 ± 1.5 (lys, pork sausage).

### 3.4. Digestible Indispensable Amino Acid Reference Ratio and Score

**Figure 2:**
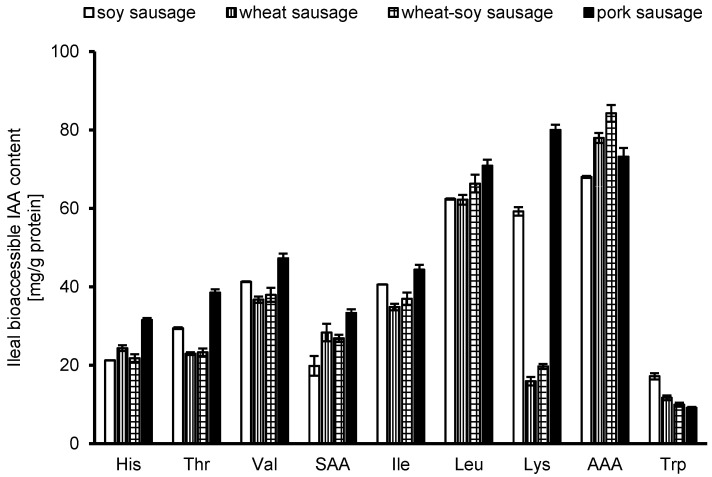
The concentration of bioaccessible indispensable amino acids (IAAs) in mg/g protein, measured after ileal digestion, is shown. The analysis covers histidine (His), threonine (Thr), valine (Val), isoleucine (Ile), Leucine (Leu), lysine (Lys), and tryptophan (Trp). Sulfur amino acids (SAAs) are reported as the sum of cysteine and methionine, and aromatic amino acids (AAAs) as the sum of tyrosine and phenylalanine. Results represent the mean values (*n* = 3) ± SD for each sausage formulation.

The ileal bioaccessible IAA content was quantified in all products (Figure 2). The aromatic IAAs (tyrosine and phenylalanine) showed the highest values, while tryptophan has the lowest values, likely due to the comparably low tryptophan content in all products (Table 1). Lysine levels showed considerable variation among the products. While soy and pork sausages had roughly 3 to 4 times more lysine than the wheat-containing sausages, and wheat sausages contained less lysine overall (~0.5 g/100 g vs. ~0.8 and 1.1 g/100 g in the others, Table 1), this difference in total content does not completely account for the larger disparity in ileal bioaccessible IAA. The more pronounced difference in the bioaccessible fraction suggests that lysine from wheat has inherently lower bioaccessibility (see below).

To determine the DIAA reference ratio and DIAAS, the amounts of bioaccessible protein were calculated using the pattern for older children, adolescents, and adults, as defined by the FAO [6]. Table 3 displays the ratio between the reference pattern and the individual IAA in the analyzed products. Among all products, only the pork sausage achieved DIAAS values exceeding 100% for all IAAs. In the soy sausage, sulfuric amino acids (sum of methionine and cysteine) were the only limiting group, with a DIAAS of 86%. In the wheat-containing sausage, threonine and valine were slightly below 100%, with DIAAS values ranging from 92% to 95%. However, as expected, lysine showed the lowest DIAAS value (33% and 41%), reflecting its low ileal bioaccessible lysine content. The nutritional quality of the wheat-containing products was limited, with DIAAS values for lysine falling well below the FAO threshold of 75% for good protein quality [6], indicating an insufficient supply of this IAA.

### 3.5. The Antioxidant Potential of the Bioaccessible Fraction

**Figure 3:**
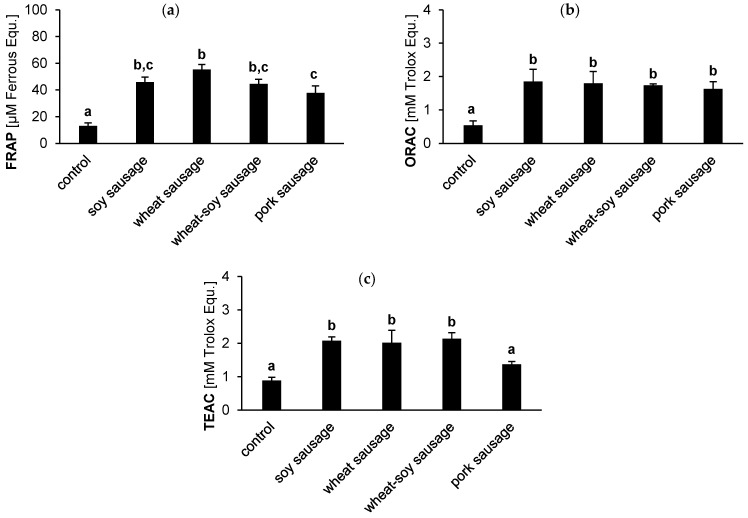
Antioxidant activity (FRAP (**a**), ORAC (**b**), and TEAC (**c**)) in the bioaccessible fraction of sausages. Results compare soy, wheat, wheat-soy, and pork sausages with a blank digestion sample (control). Values are presented as the mean (*n* = 3) ± SD, quantified in μmol/L Ferrous equivalents (**a**) or mmol/L Trolox equivalents (**b**,**c**). Distinct letters highlight a statistically significant difference between the tested products (Tukey’s test, *p* < 0.05).

The antioxidant activity of the sausage bioaccessible fractions was analyzed using FRAP, ORAC, and TEAC assays. Although the assays are based on different measurement principles, the outcomes are comparable (Figure 3). The plant-based sausages consistently showed high antioxidant capacities across all metrics. However, the superior activity compared to the pork sausage was assay-dependent. While all plant-based sausages exhibited a significantly higher antioxidant potential than the pork sausage in the TEAC assay (Figure 3c), this was not observed across the other metrics. Specifically, in the FRAP and ORAC assays, the soy and wheat-soy sausages showed no significant difference compared to the pork sausage (Figure 3a,b). In FRAP, only the wheat sausage demonstrated a significantly higher value than the pork counterpart. Overall, pork sausages generally showed the lowest values, with statistically significant differences observed against at least one plant-based variant in two out of three assays (FRAP and TEAC). These findings underscore that the overall antioxidant advantage of plant-based sausages is strongly influenced by the specific assay employed and is not uniform across all formulations.

## 4. Discussion

The aim of this study was to evaluate the protein nutritional quality, using DIAAS, and to assess the antioxidant capacity of the bioaccessible fraction following in vitro digestion, comparing scalded sausages made from soy, wheat, and a wheat-soy blend with a traditional sausage made from pork.

A DIAAS value of 100% or higher indicates that the protein source meets or exceeds the IAA requirements for human nutrition, classifying it as a high-quality protein [6]. In the present study, the pork sausage exhibited a DIAAS of 116%, thereby meeting the FAO’s criteria for high-quality protein [6]. The soy-based sausage achieved a DIAAS of 86%, meeting the threshold for good protein quality (DIAAS between 75% and 99%). Notably, all IAAs in the bioaccessible fraction of the soy sausage exceeded 100%, except for the sulfur-containing amino acids (the sum of methionine and cysteine), which are commonly the limiting amino acids in soybeans [25]. The DIAAS of 86% observed for the soy-based sausage is highly consistent with reported values for soy products [26] and soy-based burger patties [27]. This indicates that the thermal processing into a sausage emulsion did not significantly impair the protein’s inherent digestibility. In stark contrast, the low DIAAS of 33% for the wheat sausage reflects the well-known intrinsic deficiency of lysine in wheat protein [28] and cereals in general [29]. Not only did the wheat-based sausages contain less lysine per gram of protein, but the lysine digestibility was also lower compared with the other sausages, which is in line with other studies analyzing the protein content in plant-based food alternatives [19,25,30].

While combining grains and legumes is a common nutritional strategy to achieve a complete amino acid profile, the addition of soy to wheat in the mixed sausage only marginally improved the DIAAS. The mixed sausage contained 21% isolated wheat protein and 29% tofu (Appendix A, Table A1), contributing approximately 4.35 g of soy protein to the 21 g of wheat protein [31]. This combination resulted in an 8% improvement in DIAAS, suggesting that increasing the proportion of soy could further enhance the amino acid profile. Indeed, one study focusing on optimized plant protein blends suggests that a soy to wheat ratio of 90:10 is needed for optimal DIAAS values [32]. These findings underscore the importance of not only combining different plant protein sources but also optimizing their proportions to achieve a balanced and complete amino acid profile in plant-based meat alternatives [30]. While previous research has focused on the protein quality of isolates or raw plant ingredients, data on highly processed plant-based meat alternatives remains scarce in the literature. Furthermore, to the best of our knowledge, no prior study has investigated the protein quality and bioaccessibility of plant-based sausages.

The DIAAS values observed here provide a detailed and physiologically relevant assessment of the protein quality of these processed sausages. This level of precision, particularly the reliance on true ileal digestibility for individual amino acids, is a significant advantage over older methods like the Protein Digestibility Corrected Amino Acids Score (PDCAAS), which utilized fecal digestibility and suffered from truncation at 100%. The use of the dynamic tiny-TIMsg model ensures that these DIAAS values represent the most physiologically accurate prediction available for human consumption, a crucial consideration when evaluating the nutritional claims of novel food matrices.

Different types of oxidants require antioxidant defense systems that act through various strategies. Non-enzymatic food antioxidants may act at different levels in the oxidation process, e.g., by scavenging to initiate reactive oxygen species or peroxyl radicals, bind metal ions such as iron or copper, or modulate endogenous antioxidant or repair enzymes [33]. Plant-based products are known for a better antioxidative capacity compared to food of animal origin [34]. In line with these findings, these results demonstrate high antioxidative effects of all plant-based sausages (Figure 3). Plant-based antioxidants are highly effective due to their phenolic content, including flavonoids and other polyphenols, which are especially effective at scavenging free radicals and mitigating oxidative stress [35,36] (for review see [33]). Both soy and wheat are rich in phenolic compounds with antioxidant activity like isoflavones, flavonoids, and phenolic acids [37,38]. In addition, wheat and soy foods contain phytic acid which, similar to several polyphenols, such as ferulic and chlorogenic acids, can bind iron and copper ions. This protects against oxidative damage induced by these ions. All plant-based test sausages demonstrated strong antioxidative activity in FRAP, ORAC, and TEAC assays. Furthermore, some wheat and soy polyphenols, such as ferulic acid, can inhibit prooxidative enzymes such as lipoxygenase, thereby protecting against oxidative damage [39]. Although Uddin et al. reported that combining soy flour with methanolic wheat extracts enhanced antioxidant activity [40], the present study found no additional benefit from soy-wheat blending. This is probably due to the use of isolated wheat protein as an ingredient in our sample products. The isolated protein does not contain the bran and aleurone layers, which are the main sources of polyphenols such as ferulic acid [41]. Consequently, while specific phytochemicals were not quantified in this study, the superior antioxidant capacity of the plant-based sausages is hypothesized to be driven by the remaining phenolic profile inherent to the soy and wheat ingredients.

Although both raw and cooked pork generally have low intrinsic antioxidant activity, the antioxidant capacity increases significantly after digestion (assessed using ABTS and FRAP assays [42]) which is in line with the presented results (Figure 3). This increase is attributed to the release of bioactive peptides (e.g., carnosine and anserine), free amino acids (e.g., cysteine, methionine, and aromatic amino acids), and other reducing compounds generated during enzymatic hydrolysis [43,44]. Carnosine and anserine, in particular, are recognized for their strong radical scavenging and metal-chelating abilities [45]. Furthermore, other reducing compounds generated during enzymatic hydrolysis can contribute to the overall antioxidant effect in the digested fraction [46]. Moreover, herbs and spices included in all sausage formulations may have increased antioxidant activity [47]. However, metabolomic data suggest that the majority of phytochemicals derive from the main plant ingredients themselves [13]. The observed assay outcomes were shaped by both the initial ingredients and the digestion process. Since antioxidant activity was measured after in vitro digestion, the digestion process itself likely played a pivotal role, as it can enhance the bioaccessibility of phenolics and flavonoids [48,49].

Although assessing the nutritional quality of individual food items provides valuable insights, it does not adequately evaluate the nutritional sufficiency of complete meals or overall dietary patterns. A single food product cannot provide the full spectrum of essential nutrients required for optimal health. Increasingly, research highlights that the overall dietary pattern, including the combination and diversity of foods consumed over time, plays a more critical role for health than individual foods or isolated nutrients [50]. This holistic approach recognizes the synergistic interactions between nutrients and food components, which can significantly influence nutrient absorption and long-term health outcomes. However, assessing the nutritional value of individual foods still remains essential because they are the fundamental building blocks for constructing a balanced diet, identifying nutrient density, informing product development, and meeting specific nutritional needs or regulatory requirements.

This study used the dynamic, computer algorithm-controlled tiny-TIMsg model, which simulates the conditions of the human stomach and small intestine. This model has a number of food-adjusted algorithms that allow it to mimic human digestive parameters such as gradual acidification of the stomach and the neutralization in the small intestine compartment, as well as to simulate the gradual secretion of gastric and intestinal enzymes, bile, and the peristaltic and the transit time, making the model physiological.

Although the tiny-TIMsg model provides high-resolution data on amino acid bioaccessibility, it remains an in vitro simulation, e.g., this model does not include the epithelial cells with transepithelial transport systems that present in vivo. Therefore, absorption and metabolism of food constituents cannot be fully mimicked. Furthermore, tiny-TIMsg uses algorithms with standardized parameters for enzymes, pH, and other parameters, rather than taking into account the individual characteristics of the gastrointestinal tract.

Human intervention studies deliver the most physiological results. Methods used include ileostomy models and the nasojejunal technique [51]. However, these approaches may be influenced by health conditions, may be accompanied by health complications, and can only be used in a few situations [52]. Future research should therefore aim to validate these findings through human or animal in vivo trials.

Taken together, the bioaccessible fractions of the investigated plant-based sausages exhibited a higher antioxidant potential than the pork-based sausage. This is potentially attributed to their polyphenol content, a group of compounds recognized for its positive impact on health. The DIAAS in vitro method demonstrated that the highest DIAAS values were obtained for the pork sausage limited by leucine (116%), followed by the soy sausage, which was limited in sulfur-containing amino acids (86%), and the lowest DIAAS values were measured for the wheat and wheat-soy sausages, which were limited by lysine (33% and 41%, respectively). These results indicate that especially for population groups at increased risk of inadequate supply of IAAs, it is important not only to combine different plant protein sources but also to optimize their proportions to achieve a balanced and complete amino acid profile.

## Figures and Tables

**Table 1:** Amino acid residue content [g/100 g] in sausages made from soy, wheat, wheat-soy, and pork ^1^.

	Soy Sausage	Wheat Sausage	Wheat-Soy Sausage	Pork Sausage
	g/100 g Product	g/100 g Product	g/100 g Product	g/100 g Product
histidine	0.312 ± 0.020	0.760 ± 0.075	0.539 ± 0.024	0.437 ± 0.049
asparagine	0.109 ± 0.016	0.139 ± 0.017	0.092 ± 0.006	0.280 ± 0.032
serine	0.517 ± 0.021	1.113 ± 0.0143	0.999 ± 0.021	0.428 ± 0.012
glycine	0.418 ± 0.019	0.832 ± 0.087	0.691 ± 0.016	0.677 ± 0.038
arginine	0.847 ± 0.031	1.230 ± 0.091	1.059 ± 0.117	0.892 ± 0.043
aspartic acid	1.437 ± 0.063	0.977 ± 0.140	1.067 ± 0.010	1.145 ± 0.054
glutamic acid	2.259 ± 0.107	9.171 ± 1.038	6.564 ± 0.026	1.588 ± 0.071
threonine	0.438 ± 0.045	0.731 ± 0.083	0.586 ± 0.052	0.537 ± 0.024
alanine	0.495 ± 0.024	0.726 ± 0.078	0.627 ± 0.007	0.718 ± 0.038
proline	0.577 ± 0.025	3.159 ± 0.337	2.353 ± 0.039	0.596 ± 0.034
lysine	0.822 ± 0.040	0.534 ± 0.078	0.499 ± 0.036	1.075 ± 0.048
tyrosine	0.380 ± 0.028	0.924 ± 0.115	0.755 ± 0.032	0.494 ± 0.058
valine	0.624 ± 0.030	1.163 ± 0.118	0.950 ± 0.015	0.667 ± 0.033
isoleucine	0.603 ± 0.027	1.105 ± 0.116	0.921 ± 0.015	0.626 ± 0.034
leucine	0.917 ± 0.042	1.928 ± 0.201	1.611 ± 0.025	0.979 ± 0.050
phenylalanine	0.616 ± 0.025	1.477 ± 0.156	1.269 ± 0.022	0.540 ± 0.028
cysteine	0.211 ± 0.005	0.737 ± 0.122	0.528 ± 0.027	0.139 ± 0.013
methionine	0.162 ± 0.003	0.498 ± 0.069	0.360 ± 0.018	0.298 ± 0.027
tryptophan	0.229 ± 0.012	0.526 ± 0.183	0.259 ± 0.035	0.135 ± 0.009
Ʃ	11.973 ± 0.583	27.731 ± 3.250	21.730 ± 0.542	12.249 ± 0.693

^1^ Content of amino acid residues—the amino acid content is corrected for the loss of one water molecule per peptide bond; values are given as mean (*n* = 3) ± SD.

**Table 2:** In vitro digestibility [%] of indispensable amino acids in sausages made from soy, wheat, wheat-soy, and pork ^1^.

	Soy Sausage	Wheat Sausage	Wheat-Soy Sausage	Pork Sausage
	Mean Value%	Mean Value%	Mean Value%	Mean Value%
Amino Acids				
His	81.7 ± 0.2	87.8 ± 2.7	87.3 ± 3.9	89.1 ± 1.4
Thr	80.7 ± 0.5	85.9 ± 1.3	85.8 ± 3.5	88.5 ± 1.9
Val	79.4 ± 0.2	86.6 ± 1.9	86.2 ± 4.1	87.5 ± 2.2
Met	74.8 ± 7.4	78.4 ± 6.0	82.0 ± 3.3	80.9 ± 2.0
Ile	80.8 ± 0.1	86.4 ± 2.0	86.5 ± 3.7	87.4 ± 2.4
Leu	81.7 ± 0.2	88.4 ± 1.8	88.8 ± 3.0	89.4 ± 2.0
Lys	86.4 ± 1.6	81.6 ± 5.5	85.1 ± 2.8	91.9 ± 1.5
Phe	83.2 ± 0.3	90.9 ± 1.2	91.8 ± 2.1	89.3 ± 2.1
Trp	76.7 ± 3.4	81.9 ± 4.2	82.7 ± 4.3	83.7 ± 1.6

^1^ Amino acid digestibility is presented as the proportion of the amino acid found in the bioaccessible fraction relative to the entire recovered amount (sum of bioaccessible and non-bioaccessible content). The individual amino acids quantified are histidine (His), threonine (Thr), valine (Val), methionine (Met), isoleucine (Ile), leucine (Leu), lysine (Lys), phenylalanine (Phe), and tryptophan (Trp). Data are listed as mean values (*n* = 3) ± SD.

**Table 3:** DIAA reference ratio for IAAs in sausages made from soy, wheat, wheat-soy, and pork ^1^.

	DIAA Reference Ratio								
	His	Thr	Val	SAA	Ile	Leu	Lys	AAA	Trp
soy sausage	1.33	1.18	1.03	0.86	1.35	1.02	1.23	1.66	2.60
wheat sausage	1.52	0.92	0.92	1.23	1.16	1.02	0.33	1.90	1.77
wheat-soy sausage	1.36	0.93	0.95	1.17	1.23	1.09	0.41	2.06	1.50
pork sausage	1.97	1.54	1.18	1.45	1.48	1.16	1.67	1.79	1.38

^1^ The DIAA reference ratio was computed by taking the ratio of the indispensable amino acid content divided by the respective reference pattern defined by the FAO report [6] for older children, adolescents, and adults. The indispensable amino acids included in the calculation are histidine (His), threonine (Thr), valine (Val), isoleucine (Ile), leucine (Leu), lysine (Lys), and tryptophan (Trp). Sulfur-containing amino acids (SAAs) were summed (cysteine + methionine), as were the aromatic amino acids (AAAs: tyrosine + phenylalanine).

## Data Availability

The original contributions presented in the study are included in the article, further inquiries can be directed to the corresponding author.

## Abbreviations

The following abbreviations are used in this manuscript:

AAA	Aromatic Amino Acid
AAPH	2,2′-Azobis(2-amidinopropane) Dihydrochloride
ABTS	2,2′-azino-bis(3-ethylbenzothiazoline-6-sulfonic acid) Diammonium Salt
DIAAS	Digestible Indispensable Amino Acid Score
FAO	Food and Agriculture Organization
FRAP	Ferric Reducing Ability of Plasma
HPLC	High-Performance Liquid Chromatography
IAA	Indispensable Amino Acid
ORAC	Oxygen Radical Absorbance Capacity
SAA	Sulfuric Amino Acid
TEAC	Trolox Equivalent Antioxidative Capacity
TIM	TNO Gastrointestinal Model
TNO	The Netherlands Organization for Applied Scientific Research
TPTZ	2,4,6-Tri-(2-pyridyl)-s-triazine

## Appendix A

**Table A1:** Ingredient list for the products as given on the package.

Product	Ingredients
**Tofiner**	Tofu 75% (soya beans 55%, water, coagulating agents: magnesium chloride, calcium sulfate), cold-pressed sunflower oil, soya sauce (water, soya beans, wheat, sea salt), oatmeal, sea salt, thickening agent: guar gum, fenugreek, coriander, white pepper, black pepper, sweet paprika, hot paprika, caraway, nutmeg, garlic, beech wood smoke.
**Vegane Weenies**	82% seitan (water, wheat protein), high-oleic sunflower oil, yeast extract, rock salt, spices (contains mustard), onions, thickener: locust bean gum, paprika extract, beech wood smoke.
**Tofu & Seitan Wiener**	29% tofu (soya beans, water, coagulant: magnesium chloride, calcium sulfate), water, 21% wheat protein, sunflower oil, red bell pepper, sea salt, spices, raw cane sugar, thickener: locust bean gum, tomato paste, herbs, celery
**Deutschländer**	Pork 86%, water, salt, spices, spice extracts (contains celery), dextrose, antioxidant: ascorbic acid, preservative: sodium nitrite, sheep casting, beech wood smoke.

**Table A2:** Nutrient information for the products as given on the package.

Product		Energy		Carbohydrates		Fat		Protein	Salt	Fiber
	Protein Source	[kJ/100 g]	[kcal/100 g]	Total	Sugar	Total	Saturated Fatty Acids			
Tofiner	Soy	1064	256	5.3	<0.5	19	2.6	15	1.8	
Vegane Weenies	Wheat	1123	269	3.7	<0.5	15	1.3	31	1.7	
Tofu & Seitan Wiener	Soy, Wheat	1072	257	4.1	2.9	15	1.9	26	2.2	2
Deutschländer	Pork	950	229	0.5	<0.5	19	7.2	14	1.8	

If not specified otherwise, values are given in [g/100 g].
